# Genetic Structure and Forensic Utility of 23 Autosomal STRs of the Ethnic Lao Groups From Laos and Thailand

**DOI:** 10.3389/fgene.2022.954586

**Published:** 2022-07-07

**Authors:** Khaing Zin Than, Kanha Muisuk, Wipada Woravatin, Chatmongkon Suwannapoom, Metawee Srikummool, Suparat Srithawong, Sengvilay Lorphengsy, Wibhu Kutanan

**Affiliations:** ^1^ Biological Science Program, Faculty of Science, Khon Kaen University, Khon Kaen, Thailand; ^2^ Department of Forensic Medicine, Faculty of Medicine, Khon Kaen University, Khon Kaen, Thailand; ^3^ Department of Biology, Faculty of Science, Khon Kaen University, Khon Kaen, Thailand; ^4^ School of Agriculture and Natural Resources, University of Phayao, Muang Phayao, Thailand; ^5^ Department of Biochemistry, Faculty of Medical Science, Naresuan University, Phitsanulok, Thailand; ^6^ The Biotechnology and Ecology Institute Ministry of Science and Technology, Vientiane, Laos

**Keywords:** laotian, Lao isan, strs, verifiler TM plus PCR amplification kit, Laos

## Abstract

The Lao Isan and Laotian are the major groups in the area of present-day northeastern Thailand and Laos, respectively. Several previous genetic and forensic studies indicated an admixed genetic structure of Lao Isan with the local Austroasiatic speaking groups, e.g. Khmer, whereas there is a paucity of reporting Laotian’s forensic short tandem repeats (STRs). Here, we newly generated 451 genotypes of seven Lao Isan and three Laotian populations (two Lao Lum and one Lao Thoeng) using 23 autosomal STRs embedded in Verifiler^TM^ plus PCR Amplification kit. We reported allelic frequency and forensic parameters in different dataset: combined ethnic Lao groups, combined Lao Isan populations and combined Laotians. Overall, the forensic parameter results indicate that this set of STRs is suitable for forensic investigation. The anthropological results revealed the genetic homogeneity of Tai-Kadai speaking Lao groups from Thailand and Laos, consistent with previous studies, while the Austroasiatic speaking groups from southern Laos showed genetic relatedness to both Lao Isan and Khmer. In sum, STRs allelic frequency results can provide the genetic backgrounds of populations which is useful for anthropological research and also strengthens the regional forensic database in both countries.

## Introduction

As lands with much common history, the areas of present-day Laos and Thailand share multiple cultural and historical perspectives (Arnamwat, 1985; Teerawit, 2001) and a border delineated by high mountains and the Mekong River. With a geography that encompasses both upland and lowland areas located in the heart of Mainland Southeast Asia (MSEA), both countries are served with land suitable for human occupations and harbor multiple diverse ethnolinguistic groups. With population sizes of ∼6.86 million in Laos and 68.62 million in Thailand (Eberhard et al., 2020), there are 85 and 70 different languages in Laos and Thailand, respectively. All of these languages are classified as belonging to five major language families: Tai-Kadai (TK), Austroasiatic (AA), Sino-Tibetan (ST), Hmong-Mien (HM) and Austronesian (AN). The most common language family is the TK language with ∼4.29 million speakers in Laos and 46.69 million in Thailand, while AA is the second most commonly spoken language in Laos (∼1.71 million) and Thailand (∼2.07 million). However, the number of AA languages is higher (49 in Laos and 26 in Thailand) than TK languages (20 in Laos and 16 in Thailand), reflecting greater diversification of AA than TK. The ST and HM languages are less spoken in both countries (ST: 11 in Laos and 19 in Thailand; HM: 4 in Laos and 3 in Thailand) while the AN family is restricted to southern Thailand (6 languages) (Eberhard et al., 2020).

In Laos, geographic criteria are generally used to grouping populations; the major TK speaking Laotians or Lao Lum (∼4.3 million) inhabit in the lowland with various dialects spoken, e.g., Luang Prabang, Vientiane and Savannakhet (Eberhard et al., 2020). There are ∼1.7 million AA speaking Laotians or Lao Thoeng which refers to mid-landers or uplanders, while ∼0.2 million of the HM and ST speaking Laotians are highlanders known as Lao Sung (Schliesinger, 2003; Eberhard et al., 2020). In Thailand, the major TK speaking groups in four different regions are known as Khonmueang in the North, Lao Isan in the Northeast, Central Thai in the Central, and Southern Thai or Khon Tai in the South. Among those four major Thai groups, Lao Isan is ethnically closer to Laotian. Lao Isan are ethnically Lao but citizens of Thailand; they were historically relocated from Laos during the 14th to 18th century A.D. (Vallibhotama, 1989; Myers, 2005; Mishra, 2010). Both Laotian and Lao Isan are regarded as the ethnic Lao group which makes up ∼62.53% of the total population in Laos and a major group, with a population of ∼15 million, in the northeast of Thailand (Rakow, 1992; Schliesinger, 2003; Schlemmer, 2017; Eberhard et al., 2020).

Beside the Lao Isan, northeastern Thailand especially in the lower part is home to ∼1.4 million AA-speaking Khmer people (Eberhard et al., 2020) and ∼400,000 AA-speaking Kuy or Suay people who are presently trilingual, speaking both Khmer and Lao in addition to Kuy language (Premsrirat, 1997; Eberhard et al., 2020). Numerous archaeological sites since around 6^th^ century A.D. evidently attest that the Khmer are native to present-day northeastern Thailand prior to the arrival of the Lao (Coedes, 1968) whereas the Kuy migrated from southern Laos to northeastern Thailand around the 17th to 18th century A.D. (Sa-ard, 1984). Generally speaking, the AA-speaking Kuy in Laos nowadays are also one of the Lao Thoeng groups.

Several previous genetic studies of Laotian and Lao Isan were based on mitochondrial (mt) DNA and Y chromosome (Bodner et al., 2011; Kutanan et al., 2014; Kutanan et al., 2017; Kutanan et al., 2019; Kutanan et al., 2021) and genome-wide data (McColl et al., 2018; Tätte et al., 2019; Kutanan et al., 2021). However, data from forensic microsatellites or short tandem repeats (STRs) has been much less published, especially with populations from Laos (Srithawong et al., 2015, 2020). Furthermore, previous STRs data were based on 15 autosomal loci. However, more STRs can increase resolution for complex paternity cases, e.g., determining the true relationship between parent-child, siblings or half sibling (O’Connor et al., 2010; Alsafiah et al., 2019) and enhance the discrimination power in cases of partial DNA profiles and DNA mixtures (Haidar et al., 2021). Several forensic kits have been developed to expand the number of STR markers required for the Combined DNA Index System (CODIS) and for the European Standard Set (ESS) (Gill et al., 2006; Hares, 2015), e.g., VeriFiler Plus PCR Amplification Kit (Applied Biosystems, United States) which is a six-dye kit that can amplify 23 autosomal STR loci (*D3S1358, vWA, D16S539, CSF1PO, TPOX, D8S1179, D21S11, D18S51, D2S441, D19S433, TH01, FGA, D22S1045, D5S818, D13S317, D7S820, D10S1248, D1S1656, D12S391, D2S1338*, *D6S1043, Penta D, Penta E*), 1 insertion/deletion polymorphic marker on the Y chromosome (Y indel) and Amelogenin (sex-determining marker). This kit has already been validated following SWGDAM (Scientific Working Group on DNA Analysis Methods) guidelines (Al Janaahi, 2019; Alsafiah, 2019; Alsafiah et al., 2019; Green et al., 2021). This present study newly generated the genetic data of 451 Lao Isan from northeastern Thailand and Laotian from Laos, using the battery of VeriFiler Plus PCR Amplification Kit. We also reported population genetics results and established allelic frequency of 23 autosomal STRs which is beneficial for future forensic investigation in Thailand and Laos.

## Materials and Methods

### Samples Collection and Extraction

A total 451 genotypes that were newly generated for 23 autosomal STR loci belonged to ten populations: seven Lao Isan and three Laotian (two Lao Lum and one Lao Thoeng). Buccal swab samples of 39 volunteers from one Laotian population were newly collected from Savannakhet Province, southern Laos; this Austroasiatic-speaking Lao population (LA1) is called Lao Thoeng. To recruit samples, we interviewed volunteers to include subjects who were unrelated for at least two generations and then collected buccal samples with informed consent. Genomic DNA was extracted using the Gentra Puregene Buccal Cell Kit (Qiagen, Hilden, Germany) according to the manufacturer’s instructions. An additional 412 genomic DNA samples from other Laotian and Lao Isan populations were retrieved from previous studies (Kutanan et al., 2017; Srithawong et al., 2020; Kutanan et al., 2021) (Table 1). Ethical approval for this study was provided by Khon Kaen University for Lao Isan and Naresuan University for Laotian.

**TABLE 1:** General information of the studied populations, genetic diversity indices and forensic parameters.

Ethnicity	Code	Sample size	Location	Linguistic classification	Average *H*_*E*_	Total allele	Gene Diversity (SD)	CMP	CPE	Loci Departed from HWE
Laotian	LA1	39	Savannakhet, southern Laos	Austroasiatic	0.7867	182	0.7811 (0.3890)	3.5854 × 10^−24^	0.999999999902770	*PentaE*
Laotian	LA2	46	Luang Prabang, northern Laos	Tai-Kadai	0.7861	199	0.7853 (0.3895)	6.0225 × 10^−25^	0.999999998620348	
Laotian	LA3	88	Vientiane, central Laos	Tai-Kadai	0.7962	213	0.7886 (0.3890)	1.2984 × 10^−26^	0.999999998315516	
Lao Isan	LAO1	41	Roi-Et, northeastern Thailand	Tai-Kadai	0.7928	197	0.7878 (0.3912)	6.5469 × 10^−25^	0.999999967018448	
Lao Isan	LAO2	45	Buriram, northeastern Thailand	Tai-Kadai	0.7901	198	0.7884 (0.3911)	5.2816 × 10^−25^	0.999999969814718	
Lao Isan	LAO3	41	Ubon Ratchathani (1), northeastern Thailand	Tai-Kadai	0.7859	200	0.7763 (0.3858)	7.8522 × 10^−25^	0.999999996073971	
Lao Isan	LAO4	48	Chaiyaphum, northeastern Thailand	Tai-Kadai	0.7946	212	0.7938 (0.3934)	1.7946 × 10^−25^	0.999999999331283	
Lao Isan	LAO5	41	Loei, northeastern Thailand	Tai-Kadai	0.7812	182	0.7782 (0.3867)	3.1171 × 10^−24^	0.999999999972446	*D5S818*
Lao Isan	LAO6	28	Ubon Ratchathani (2), northeastern Thailand	Tai-Kadai	0.7841	179	0.7792 (0.3894)	2.704 × 10^−23^	0.999999999798795	
Lao Isan	LAO7	34	Nakhon Ratchasima, northeastern Thailand	Tai-Kadai	0.7912	188	0.7889 (0.3928)	2.0605 × 10^−24^	0.999999999339757	

### DNA Amplification and STR Genotyping

We amplified 23 autosomal STR loci of all genomic samples using Verifiler^TM^ plus PCR amplification kit in a reaction volume of 25 µl with 5 µl of VeriFiler™ Plus PCR Master Mix (Applied Biosystems), 2.5 µl of the primer mix, and the remaining 17.5 µl composed of DNA template and water to adjust the DNA input amount to reach 500 pg. Amplification process was carried out on GeneAmp™ PCR System 9700 in the following conditions; 95°C for 1 min; 2 cycles: 96°C for 10 s, 62°C for 90 s; 27 cycle: 96°C for 10 s, 59°C for 90 s; 60°C for 5 min; 4°C for ∞. The PCR products were genotyped by multi-capillary electrophoresis in an ABI 3500 genetic analyzer (Applied Biosystems). The genotyping data were analyzed by Gene Mapper software v.3.7 (Applied Biosystem).

### Statistical Analyses

Arlequin v.3.5.2.2 (Excoffier and Lischer, 2010) was used to calculate allele frequency, Hardy–Weinberg equilibrium (HWE) *p* values, observed heterozygosity (*H*_*O*_), expected heterozygosity (*H*_*E*_), total alleles, and gene diversity (GD). Significant levels for the HWE were adjusted according to the sequential Bonferroni correction (α = 0.05/23) (Rice, 1989). We used *STRAF* (http://cmpg.unibe.ch/shiny/STRAF/), an online tool for STR data analysis (Gouy and Zieger, 2017), to compute several forensic parameters, i.e., power of discrimination (PD), matching probability (MP), polymorphic information content (PIC), power of exclusion (PE), and typical paternity index (TPI).

Among all 10 Lao Isan and Laotian populations, we first computed a genetic distance matrix based on number of different alleles (*F*_*st*_) using Arlequin, then plotting a matrix in three dimensions by means of multidimensional scaling (MDS) using Statistica v.10 demo (StatSoft, Inc, United States). The heatmap visualization of *F*_*st*_ and MDS values were obtained using R package (R Development Core Team). To delineate cryptic population structure, a model-based cluster analysis was investigated by STRUCTURE 2.3.4 under the following prior parameters: admixture, correlated allele frequencies, and assistance of sampling locations (LOCPRIOR model) (Pritchard et al., 2000; Falush et al., 2003; Hubisz et al., 2009). We ran ten replications for each cluster (K) from 2 to 10 with burn-in length of 100,000 iterations followed by 200,000 iterations. The STRUCTURE outputs were merged to compute a second-order rate of change logarithmic likelihood between subsequent *K* values (*△K*) (Evanno et al., 2005) by STRUCTURE Harvester (Earl and vonHoldt, 2012) in order to discover the ideal *K* value in the data. CLUMPAK was used to construct a single-set result from 10 replications of STRUCTURE outputs to validate the dynamic approach determining the optimal similarity threshold for each value of *K* (Kopelman et al., 2015); CLUMPAK outputs were graphically modified by DISTRUCT (Rosenberg, 2003).

**FIGURE 1:**
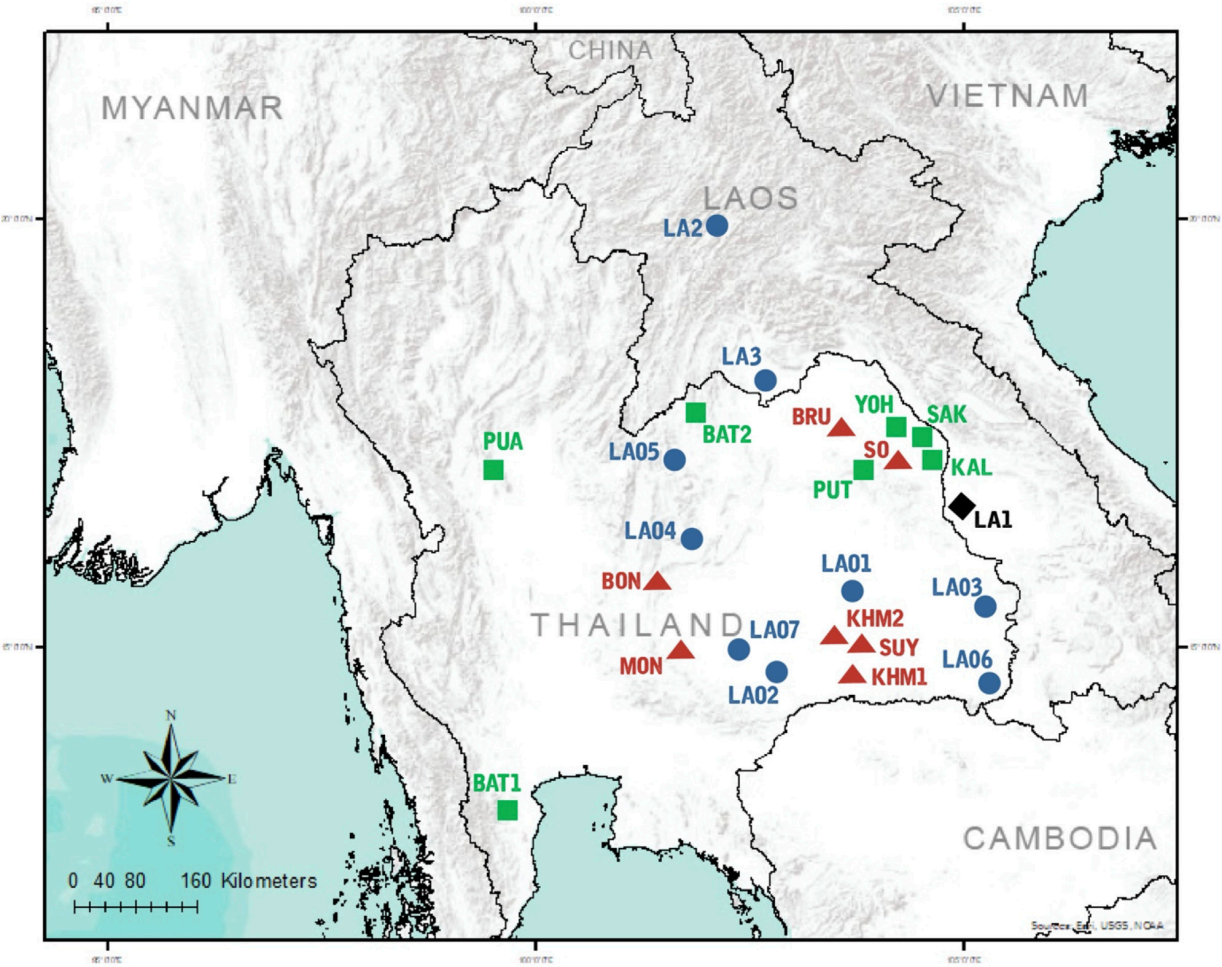
Map of sampling locations. 10 populations were sampled in the present study, combined with data from 14 populations collected in previous studies. Population codes are given in .Supplementary Table S16 Black diamond, blue circles, green squares, and red triangles represent AA-speaking Laotian, ethnic Lao group, compared TK-speaking and AA-speaking populations respectively.

To reveal population relationships with other Thai populations, we collected previously published data of 15 STRs from 14 populations: BlackTai1, BlackTai2, Phutai, Phuan, Seak, Nyaw, Kaleang, Bru, Nyahkur, Mon, Soa, Khmer1, Khmer 2 and Suay (Srithawong et al., 2015; Chantakot et al., 2017; Srithawong et al., 2020). When the newly generated data of Lao Thoeng were combined with this published data, it provided a total raw 15 STRs genotype data of 1,039 samples belonging to 24 populations for subsequent analyses (Figure 1). As mentioned previously, the same software and parameters were employed to estimate genetic distances and genetic structure.

Mantel test (Mantel, 1967) in Arlequin was used to estimate correlation between distance matrices of genetic vs. geography. Geographic distances (in form of great-circle distances) in Km between the approximate locations of each population were calculated from their latitudinal and longitudinal coordinates using an online tool (http://onlineconversion.com/map-greatcircle-distance.htm).

To get a more comprehensive picture of population linkages in Asia, we employed POPTREE v.2 (Takezaki et al., 2014) to build a neighbor-joining tree (NJ) based on *F*_*st*_ computation by allele frequency from 13 STRs with publicly accessible data from relevant populations (Brinkmann et al., 2002; Kim et al., 2003; Seah et al., 2003; De Ungria et al., 2005; Dobashi et al., 2005; Tie et al., 2006; Maruyama et al., 2008; Zhu et al., 2008; Untoro et al., 2009; Song et al., 2010; Kutanan et al., 2011; Yang et al., 2013; Zhai et al., 2014; Huang et al., 2015; Kutanan et al., 2015; Srithawong et al., 2015; Zhang, 2015a; Zhang, 2015b; Chantakot et al., 2017; Guo, 2017; Guo et al., 2017; Srithawong et al., 2020; Mawan et al., 2021).

## Results and Discussion

### Genetic Diversities and Forensic Parameters

A total of 451 individual raw genotypes are provided in Supplementary Table S1. The allelic frequency table of 23 STR loci in 10 individual studied populations are reported in Supplementary Table S2–S11. Hardy–Weinberg equilibrium (HWE) tests showed no significant deviation from expected values for all 23 loci (*p* > 0.05) after Bonferroni adjustment (0.05/23 = 0.002) in exception with *PentaE* in LAO1 and *D5S818* in LAO5 (Table 1). The genetic diversity indices and forensic parameters, including average *H*_*E*_, total alleles, gene diversity (GD), forensic parameters; combined matching probability (CMP), combined power of exclusion (CPE) are presented in Table 1. The average *H*_*E*_ is greater than 0.7 in all studied populations, ranging from 0.7962 (LA3) to 0.7812 (LAO5). Total number of alleles is highest in LA3 (213 alleles) and lowest in LAO6 (179 alleles). The GD ranges from 0.7763 ± 0.3858 in LAO3 to 0.7938 ± 0.3934 in LAO4.

The allelic frequency of 23 STR loci in the ethnic Lao groups (*n* = 412), comprising two Lao Lum (LA2-3) from Laos and seven Lao Isan (LAO1-7) from northeastern Thailand, is presented in Supplementary Table S12. Only two loci (*D10S1248, TPOX*) departed from the HWE (*p* > 0.05) after Bonferroni adjustment (0.05/23 = 0.002) (Supplementary Table S12). There are total 292 alleles, with the range of 8 alleles at *D16S539* and *D5S818* loci to 23 alleles at *FGA* locus and allelic frequency ranging from 0.0012 to 0.6163. Among the tested loci, *FGA* was the most polymorphic and discriminative locus with highest *H*_*E*_ (0.8821), *H*_*O*_ (0.9005), PIC (0.8699), TPI (5.0244), MP (0.0276), PD (0.9724) and PE (0.7964) with a combined power of discrimination (CPD) value of 0.999999999999999 and a CPE value of 0.999999995430163. The least polymorphic and discriminative locus was *TPOX* with the lowest *H*_*E*_ (0.5629), *H*_*O*_ (0.5122), PIC (0.5163), TPI (1.0250), MP (0.2391), PD (0.7609), PE (0.1984) (Supplementary Table S12).

We also reported the allele frequency based on the 23 STR loci of the combined Lao Isan (LAO1-7: n = 278) (Supplementary Table S13) and combined Laotian (LA1-3: n = 173) data (Supplementary Table S14). In Lao Isan dataset, there are 278 alleles varied from 8 alleles (*D16S539* and *D5S818*) to 23 alleles (*FGA*) (Supplementary Table S13) with the allelic frequency ranging from 0.0018 to 0.6025 and only one locus (*D10S1248*) departed from HWE after Bonferroni correction (0.05/23 = 0.002). Among the tested loci, *FGA* was the most polymorphic and discriminative locus with highest *H*_*E*_ (0.8837), *H*_*O*_ (0.8957), PIC (0.8712), TPI (4.7931), MP (0.0275), PD (0.9725) and PE (0.7866) with a CPD value of 0.999999999999999 and a CPE value of 0.999999994092542. The least polymorphic and discriminative locus was *TPOX* with the lowest *H*_*E*_ (0.5759), *H*_*O*_ (0.5324), PIC (0.5280), TPI (1.0692), MP (0.2275), PD (0.7725), PE (0.2174) (Supplementary Table S13).

In the combined Laotian (LA1-3) dataset, all of loci are in agreement with HWE even after Bonferroni correction. The total number of alleles is 233, varying from 6 alleles at *D3S1358, D16S539, TPOX* to 19 alleles at *FGA* and allelic frequency ranging from 0.0029 to 0.6170 (Supplementary Table S14). Among the tested loci, *FGA* was the most polymorphic and discriminative locus with highest *H*_*E*_ (0.8844), *H*_*O*_ (0.9017), PIC (0.8706), TPI (5.0882), MP (0.0314), PD (0.9686) and PE (0.7990) with a CPD value of 0.999999999999999 and a CPE value of 0.999999998686772. The least polymorphic and discriminative locus was *TPOX* with the lowest *H*_*E*_ (0.5633), *H*_*O*_ (0.5118), PIC (0.5143), TPI (1.0241), MP (0.2432), PD (0.7568), PE (0.1980) (Supplementary Table S14).

This study provides additional forensic STR loci of Lao Isan and Laotian populations. After applying Bonferroni’s correction, there was absence of departure from Hardy–Weinberg equilibrium tests in several datasets (Supplementary Table S2–S4, S6–S8, S10–S11, S14), implying that the samples are representative and the data is credible. When the PD was larger than 0.80, a STR locus was considered highly polymorphic (Shriver et al., 1995). In the ethnic Lao dataset all loci except *TPOX* had a PD value of more than 0.80 (Supplementary Table S12, S13) while in the pooled Laotian populations, all other markers were found to be highly discriminative except for *D3S1358* and *TPOX,* which showed PD values lower than 0.80 (Supplementary Table S14). All populations had high heterozygosity (average *H*_*E*_ greater than 0.7) and high CPD values (more than 0.999999999999999) which reflects the high discriminatory power of these 23 loci. In addition, the CPE values were greater than 0.9999999 which indicate that these markers are undoubtedly acceptable for paternal and maternal identification in the studied populations (Al-Eitan and Tubaishat, 2016). Likewise, the CMP in individuals improved to values ranging from 2.704 × 10^−23^ (LAO6) to 1.2984 × 10^−26^ (LA3) in this study (Table 1), when compared with the result of CMP (ranged from 2.26 × 10^−14^ to 4.16 × 10^−16^) in previous studies using AmpFLSTR Identifiler kit (Srithawong et al., 2020). As a result of using these 23 loci, this statistic implies that the chances of two people in the population having the same genetic profile are almost negligible and Verifiler^TM^ plus PCR amplification kit is an effective method for kinship analysis (Alsafiah, 2019; Alsafiah et al., 2019). The *FGA* and *TPOX* were respectively the loci showing highest and lowest TPI values in all combined datasets (Supplementary Table S12, S13, S14). Consistent with previous studies, *TPOX* was the least discriminative locus. While in this study *FGA* was the most informative locus, other studies have supported *PentaE* as the most powerful locus (Rodriguez et al., 2015; Sheng et al., 2018; Castillo et al., 2019; Chen et al., 2019; Naji et al., 2019; Shrivastava et al., 2019; Vu et al., 2021). Overall, the 23 STR loci in Verifiler^TM^ plus PCR amplification kit could be valuable for forensic investigation in Thailand and Laos.

### Genetic Affinity and Genetic Structure

**FIGURE 2:**
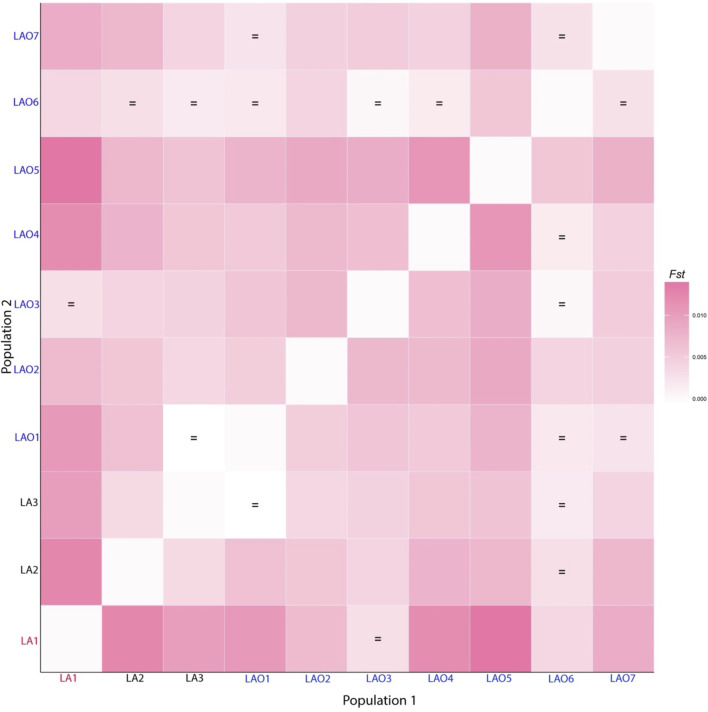
Heat plot of *F*_*st*_ values between 10 populations from Laos and Thailand. Population names are color-coded as red, black and blue representing AA-speaking Laotian, TK-speaking Laotians and Lao Isan, respectively. The “ = ” symbol indicates *F*_*st*_ values that are not significantly different from zero (*p* > 0.05).

**FIGURE 3:**
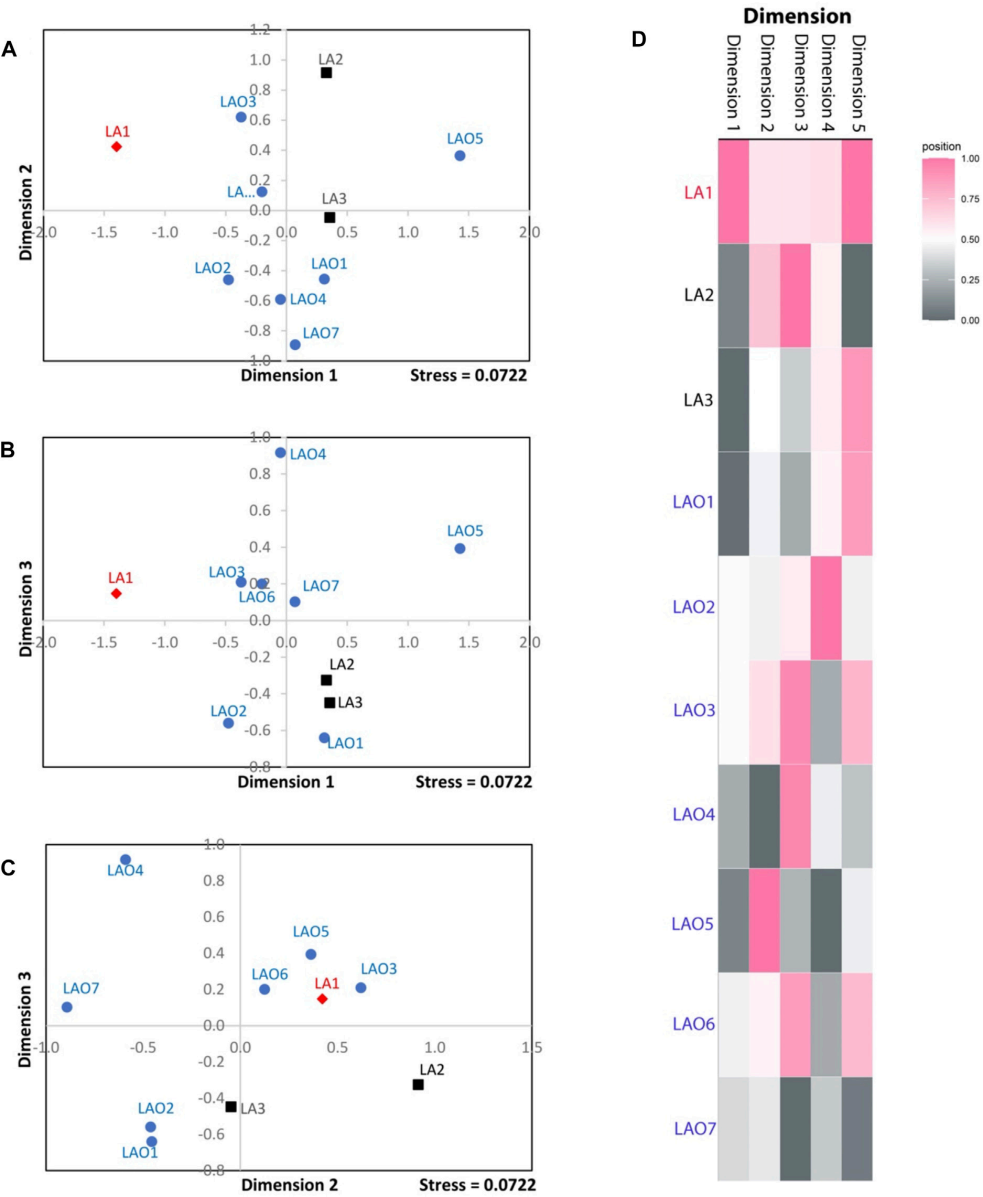
The three-dimensional MDS plot of dimension 1 vs. 2 **(A)**, 1 vs. 3 **(B)** and 2 vs. 3 **(C)** of total 10 populations. The heat plot of standardized values of MDS with five dimensions **(D)**. Red diamond, black squares, and blue circles represent AA-speaking Laotian, TK-speaking Laotians and Lao Isan populations, respectively.

**FIGURE 4:**
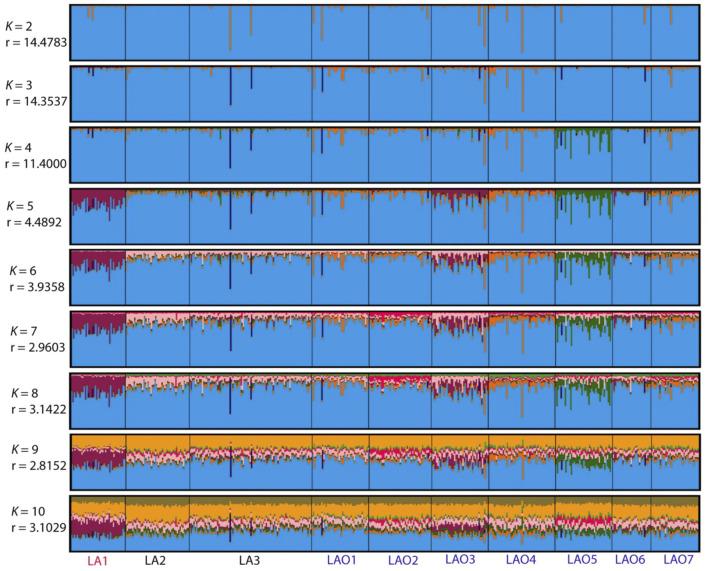
The STRUCTURE result of 10 populations from number of cluster (*K*) = 2 to 10. Each individual population is represented by a single column divided into segments whose size and colour correspond to the relative proportion of a particular cluster. Populations are separated by black lines. Population names are color-coded; red, black and blue represent AA-speaking Laotian, TK-speaking Laotians and Lao Isan populations, respectively. The parameters “*r*” indicate the informativeness of sampling location data.

To investigate genetic relationship between these 10 populations, three from Laos (LA1-3) and seven from northeastern Thailand (LAO1-7), we computed pairwise genetic distance (*F*_*st*_) based on 23 STR loci and among 45 pairwise comparisons there are 36 pairs (80%) which showed statistical differences (*p* < 0.05) (Supplementary Table S15). In general, the results of distance-based clustering methods revealed that LA1 and LAO5 showed genetic divergence from other populations (Figures 2, 3). For the model-based clustering results revealed by STRUCTURE, we ran 10 iterations of consecutive clusters (*K*) from 2 to 10. The most appropriate △*K* are *K* = 5 and 7 (Supplementary Figure S1). The LA1 showed genetic differentiation from other groups with the presence of a purple component that also saw minor emergence in LAO3 (Figure 4), indicating a certain relatedness between them. The AA-speaking LA1 and TK-speaking LAO3 speak different language but the geographic locations of these two populations are close (Figure 1); they may have contact and gene flow between them. In addition, there is a small green component in LAO5 from Loei provinces (Figure 4), reflecting the differentiation of this group, consistent with previous study (Srithawong et al., 2020).

**FIGURE 5:**
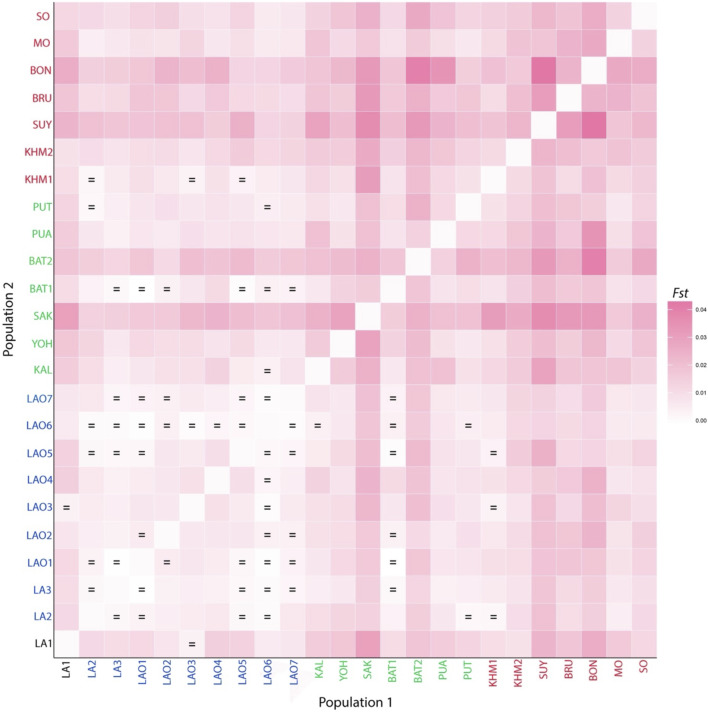
Heat plot of *F*_*st*_ values between 24 populations from Laos and Thailand. Population names are color-coded as black, blue, green and red representing AA-speaking Laotian, ethnic Lao group, compared TK-speaking and AA-speaking populations, respectively. The “ = ” symbol indicates *F*_*st*_ values that are not significantly different from zero (*p* > 0.05).

**FIGURE 6:**
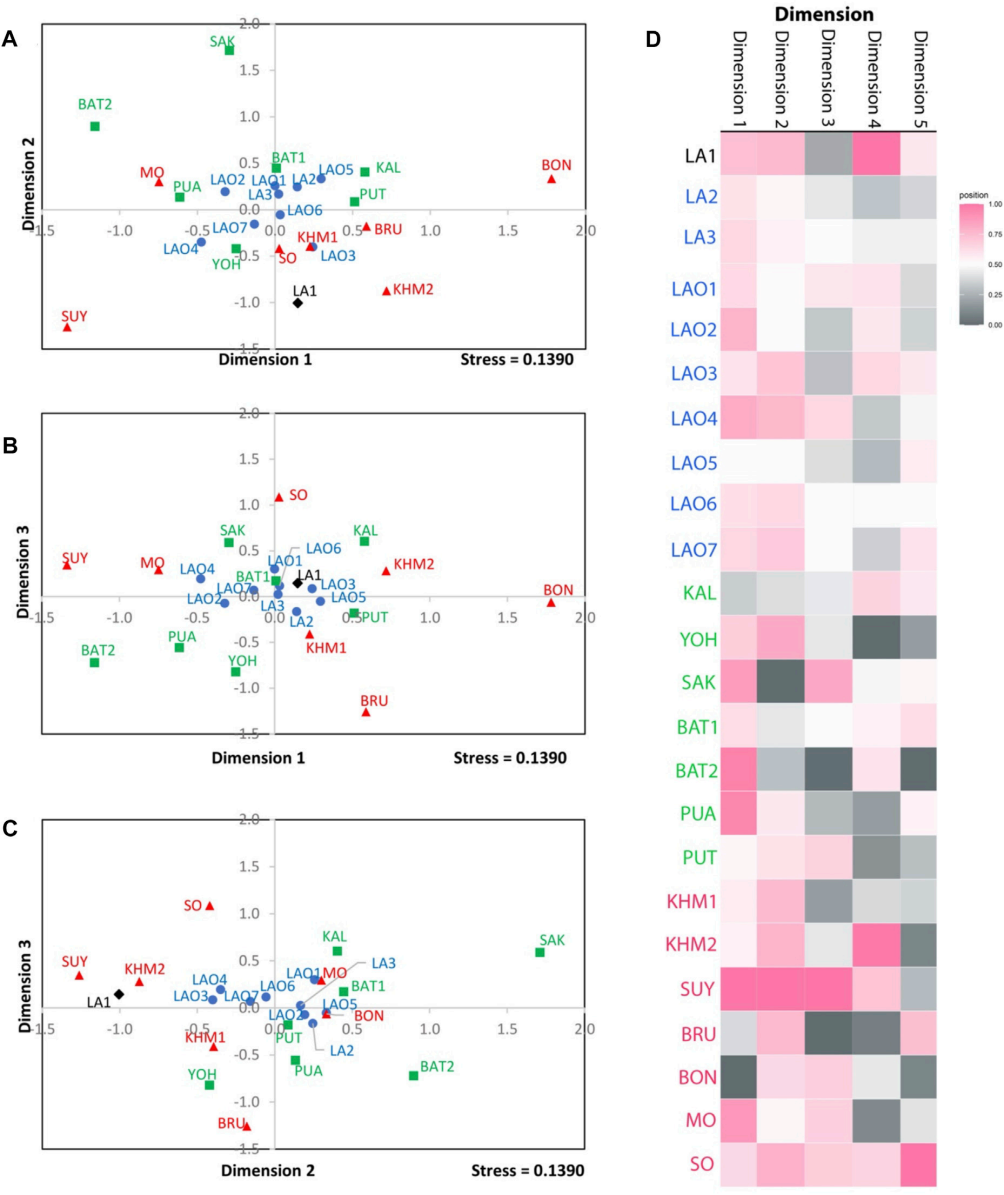
The three-dimensional MDS plots of dimension 1 vs. 2 **(A)**, 1 vs. 3 **(B)** and 2 vs. 3 **(C)** of total 24 populations. The heat plot of standardized values of MDS with five dimensions **(D)**. Black diamond, blue circles, green squares, and red triangles represent AA-speaking Laotian, ethnic Lao group, compared TK-speaking and AA-speaking populations respectively.

**FIGURE 7:**
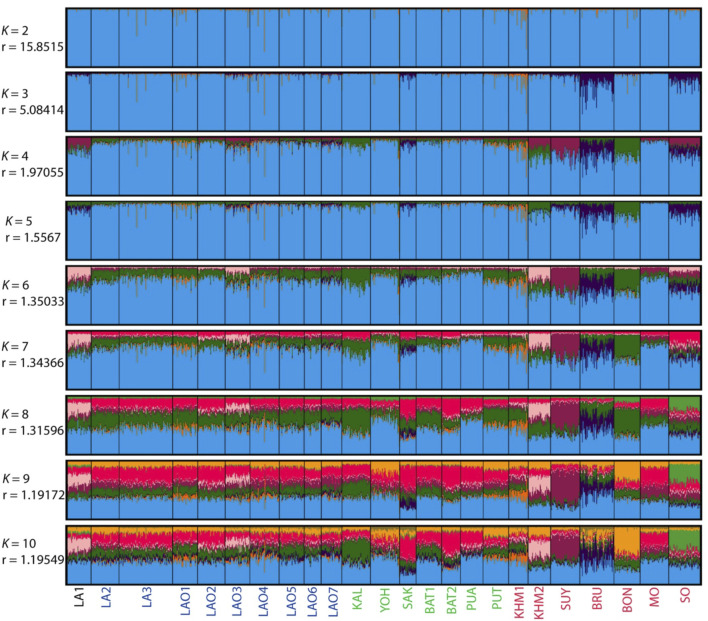
The STRUCTURE result of 24 populations from number of cluster (*K*) = 2 to 10. Each individual population is represented by a single column divided into segments whose size and colour correspond to the relative proportion of a particular cluster. Populations are separated by black lines. Population names are color-coded; black, blue, green and red represent AA-speaking Laotian, ethnic Lao group, comparing TK-speaking and AA-speaking populations, respectively. The parameters “*r*” indicate the informativeness of sampling location data.

To understand comprehensive genetic relationships among populations in northeastern Thailand and Laos, we retrieved genotypic data on 15 STRs of 14 populations from previous studies for comparison with present data (Supplementary Table S16). Again, the pairwise *F*_*st*_ value, three dimensions MDS plots and the model-based clustering STRUCTURE based on the 15 STR loci were analyzed. Among 276 pairwise comparisons of *F*_*st*_ value, there are 263 pairs (95.29%) with statistical differences (*p* < 0.05) (Supplementary Table S17). The ethnic Lao groups (LA2-3 and LAO1-7) showed a narrow range of *F*_*st*_ value, reflecting close genetic relatedness. Interestingly, with a reduced number of STRs, LA1 from southern Laos still showed significant relatedness to LAO3 from Thailand (Figure 5; Supplementary Table S17) and LAO6 showed genetic similarity to all of ethnic Lao populations (Figure 5). The MDS plots based of *F*_*st*_ value (Figures 6A–C) revealed an overall pattern of genetic homogeneity of TK speaking groups and genetic heterogeneity of AA speaking populations. The positions on the margin of MDS plots of AA-speaking LA1, BON, SUY, KHM2, SO and BRU and TK-speaking SAK and BAT2 reflect the genetic divergence of the others (Figures 6A,C,D). The STRUCTRUE results indicated that *K* = 6 is the most appropriate △*K* for describing sub-structuring of populations (Supplementary Figure S2) and at *K* = 6 all populations shared a common blue component with different proportions. In general, the AA speaking groups have reduced blue component but show additional various minor components, indicating their genetic differentiation from each other and from the TK speaking groups, which is consistent with previous mtDNA, Y chromosome and genome-wide studies (Kutanan et al., 2014; Kutanan et al., 2017; Kutanan et al., 2019; Kutanan et al., 2021). Genetic drift, isolation and population interactions with other groups are factors promoting genetic differentiations of AA speaking groups in Thailand (Kutanan et al., 2018; Kutanan et al., 2021). Interestingly, the AA-speaking LA1 and KHM2 groups and TK-speaking LAO3 share minor pink components (Figure 7), reflecting interactions among these groups. Although there is limited historical evidence supporting interactions among LA1 and KHM2, there were several reports about genetic connections between some AA-peaking populations in northeastern Thailand (Khmer, Kuy, Soa and Bru) and Lao Isan groups (Chantakot et al., 2017; Kutanan et al., 2019; 2021). Population admixture could explain a shared genetic component among these three groups.

Although some populations that lived in close geographic locations showed a certain similarity in genetic structures, e.g. LA1 and LAO3, Mantel testing showed that there were no correlations between genetic vs. geographic distances (*r* = 0.0689, *p* > 0.05 for dataset of 10 populations and *r* = -0.1570, *p* > 0.05 for dataset of 24 populations). Therefore, the overall genetic variation did not correlate with geography. In contrast to the previous study that reported correlation between mtDNA variations and geography in northeastern Thailand (Kutanan et al., 2014), this result indicates that genetic divergences between populations do not primarily influence by geography but other driving forces, e.g. genetic drift might be the probable driven factors, particularly in the AA speaking groups.

In sum, the present results indicate a genetic homogeneity of TK speaking groups in northeastern Thailand but some populations within the ethnic Lao groups exhibited their unique genetic characters, e.g. genetic distinction of LAO3 and LAO4 from the others and genetic similarity of LAO6 to other groups (Figure 5). This within-group heterogeneity was arisen from various sources as mentioned previously. We emphasize the important to study multiple samples from the same ethnic group that can provide more insights into genetic history of population.

### Asian Phylogenetic Tree

**FIGURE 8:**
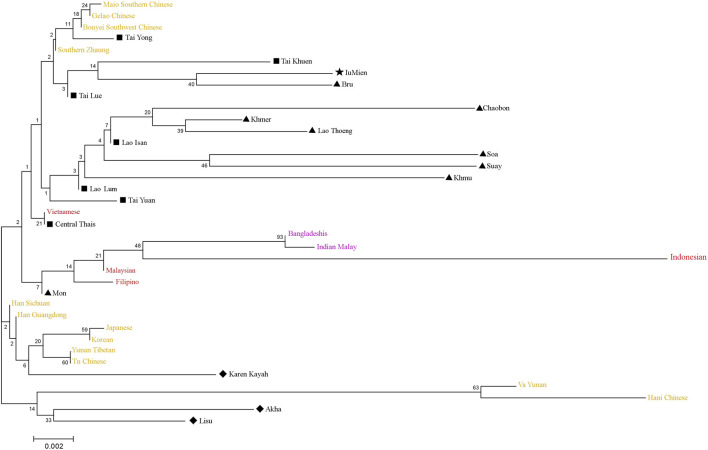
Neighbor-joining tree constructed from *F*_*ST*_ genetic distance based on the allelic frequencies of 13 STR loci among total 37 populations. The comparative populations from China/East Asia, Southeast Asia and South Asia were represented by yellow, red and purple, respectively. Populations from Thailand were indicated with black and different symbols. Diamonds, triangles, star, and squares represent ST-, AA-, HM-, and TK-speaking populations, respectively.

A neighbor-joining (NJ) tree based on *F*_*st*_ computation by allele frequency of 13 STR loci was constructed to evaluate Asian population relationships. We pooled seven Lao Isan to one group due to their close genetic relationship and likewise, the two Laotians (LA2-3) were combined to one group (Lao Lum), while the LA1 was represented by Lao Thoeng (Figure 8). The Lao Isan, Lao Thoeng and Lao Lum were clustered on the same clade with AA-speaking Chaobon, Khmer, Soa, Suay and Khmu, reflecting genetic interaction between northeastern Thai and Lao populations and AA speaking groups. Previous Y chromosomal study indicated AA ancestry in Lao Isan groups (Kutanan et al., 2019) and recent genome-wide study also supports genetic relatedness among TK-speaking groups in northeastern Thailand and Laos with AA-speaking populations, especially the Khmuic-Katuic groups (Kutanan et al., 2021).

## Conclusion

This study genotyped 23 autosomal forensic STRs using Verifiler^TM^ plus PCR amplification kit of the ethnic Lao groups from northeastern Thailand and Laos and AA-speaking Laotian from southern Laos. Although there have been some previous investigations on STRs in the region, only 15 loci were previously published. Here, we expanded the study, genotyping 23 STR loci, of which 8: *D2S441, D22S1045, D10S1248, D1S1656, D12S391, D6S1043, Penta D*, and *Penta E* were firstly reported. We generated allelic frequency table, calculated forensic parameters and investigated genetic relationships among populations. Previously there were much less forensic STRs data produced in Laos, compared to Thailand; this present study established the complete allelic frequency of STRs. Although no STRs in Laotians showed significant departure from HWE and few loci departed from HWE in other datasets, all forensic parameters indicate that this kit is suitable for forensic investigation. Genetic characterization showed that AA-speaking Laotian from southern Laos was genetically different from northern and central Lao groups who speak TK languages. In fact, the southern Laotian was more related to Lao Isan who live in vicinity. Although the Lao Isan migrated from Laos ∼200 years ago, the ethnic Lao groups (Lao Isan and TK-speaking Laotian) still showed closer genetic relatedness than the other ethnolinguistic groups, reflecting a common ancestry. In sum, the STRs allelic frequency results strengthen the regional forensic database in both countries and provide the genetic backgrounds of populations that are useful for anthropological research.

## Data Availability

The datasets presented in this study can be found in online repositories. The names of the repository/repositories and accession number(s) can be found in the article/Supplementary Material.
